# Supplementary figures and images for: Exploring the ATN classification system using brain morphology
Source: Alzheimers Res Ther. 2023 Mar 13;15:50. doi: 10.1186/s13195-023-01185-x (PMC10009950; doi:10.1186/s13195-023-01185-x)

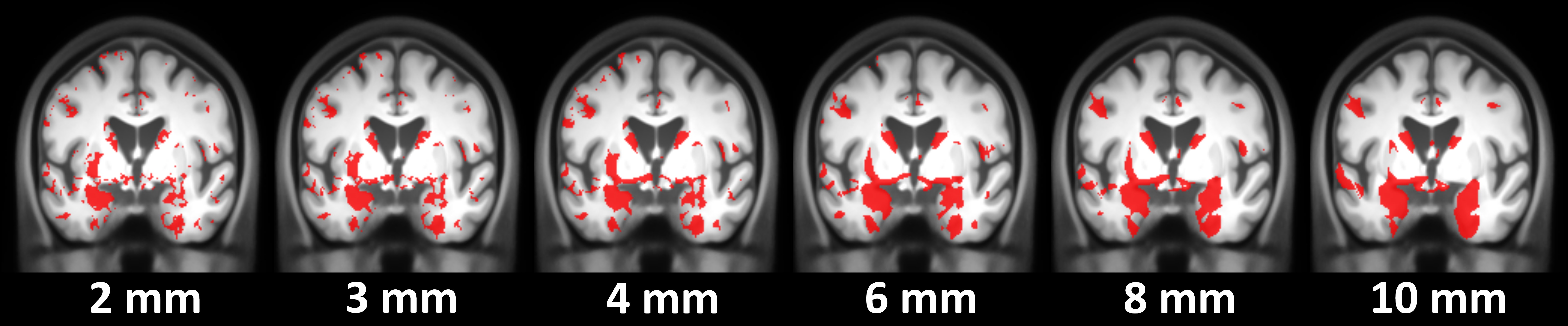

Supplement: Supplementary file 1 — Additional file 1. The impact of Gaussian blurring with different FWHM kernels sizes on model evidence for ACH. Displayed is one slice (y = 0) for each FWHM kernel size. Shown is the voxel-based evidence for monotonic volume decline of the ACH sequence (➔ A+T-N- ➔ A+T+N- ➔ A+T+N+) over 24 sequences obtained by permutation. For our model, we selected the 6 mm FWHM kernel as a compromise between reducing noise and preserving local information of volume differences. Red: voxels where sequence shows highest evidence. Neurodegeneration (N) by aHV. [file 13195_2023_1185_MOESM1_ESM.png]

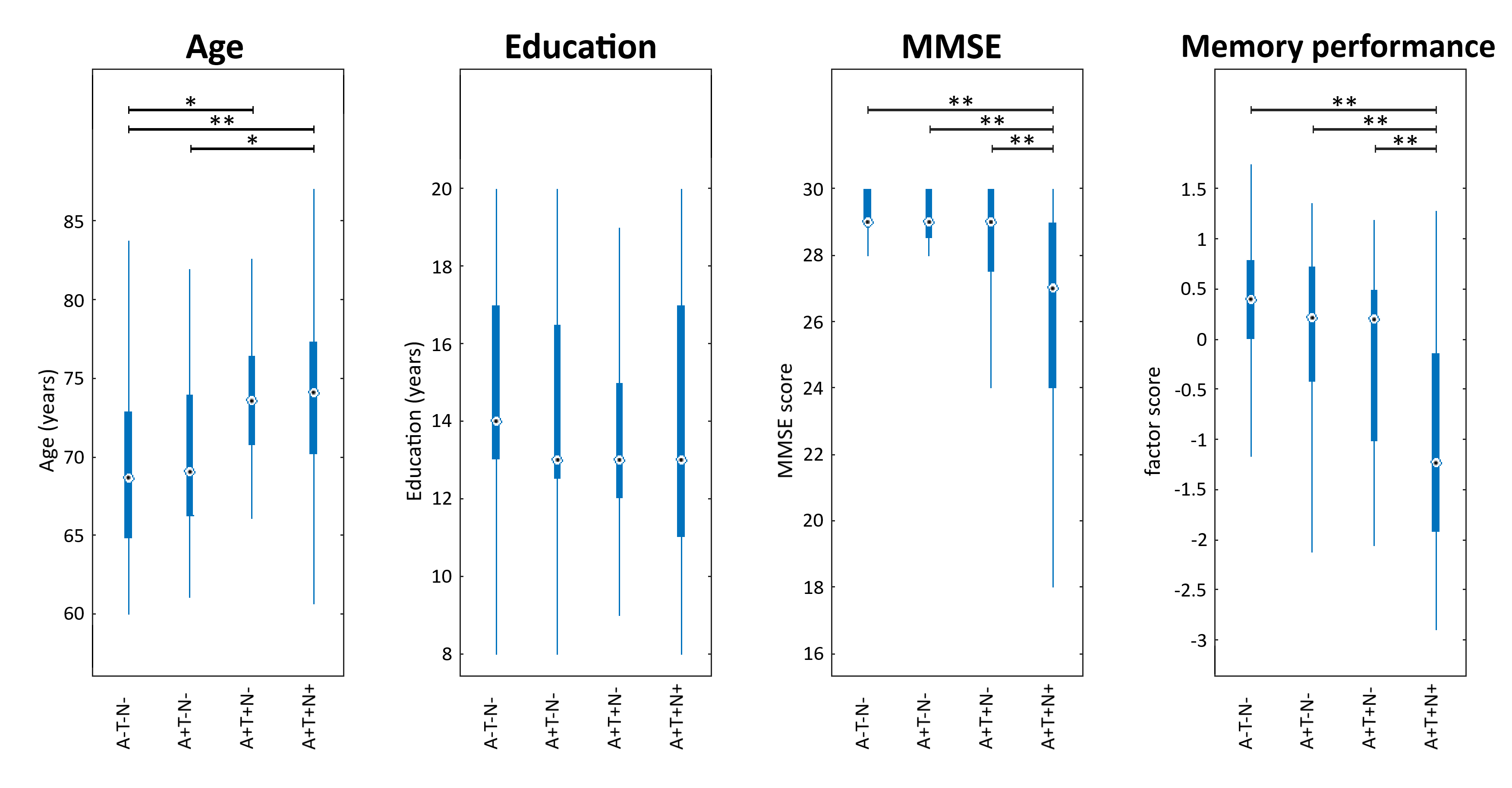

Supplement: Supplementary file 2 — Additional file 2. Comparison between selected ATN groups using CSF-total-Tau. Boxplots of age, sex, cognition for selected ATN groups. *: p < .05 after Bonferroni correction, **: p < .001 after Bonferroni correction. Neurodegeneration (N) by CSF Total Tau. [file 13195_2023_1185_MOESM2_ESM.png]

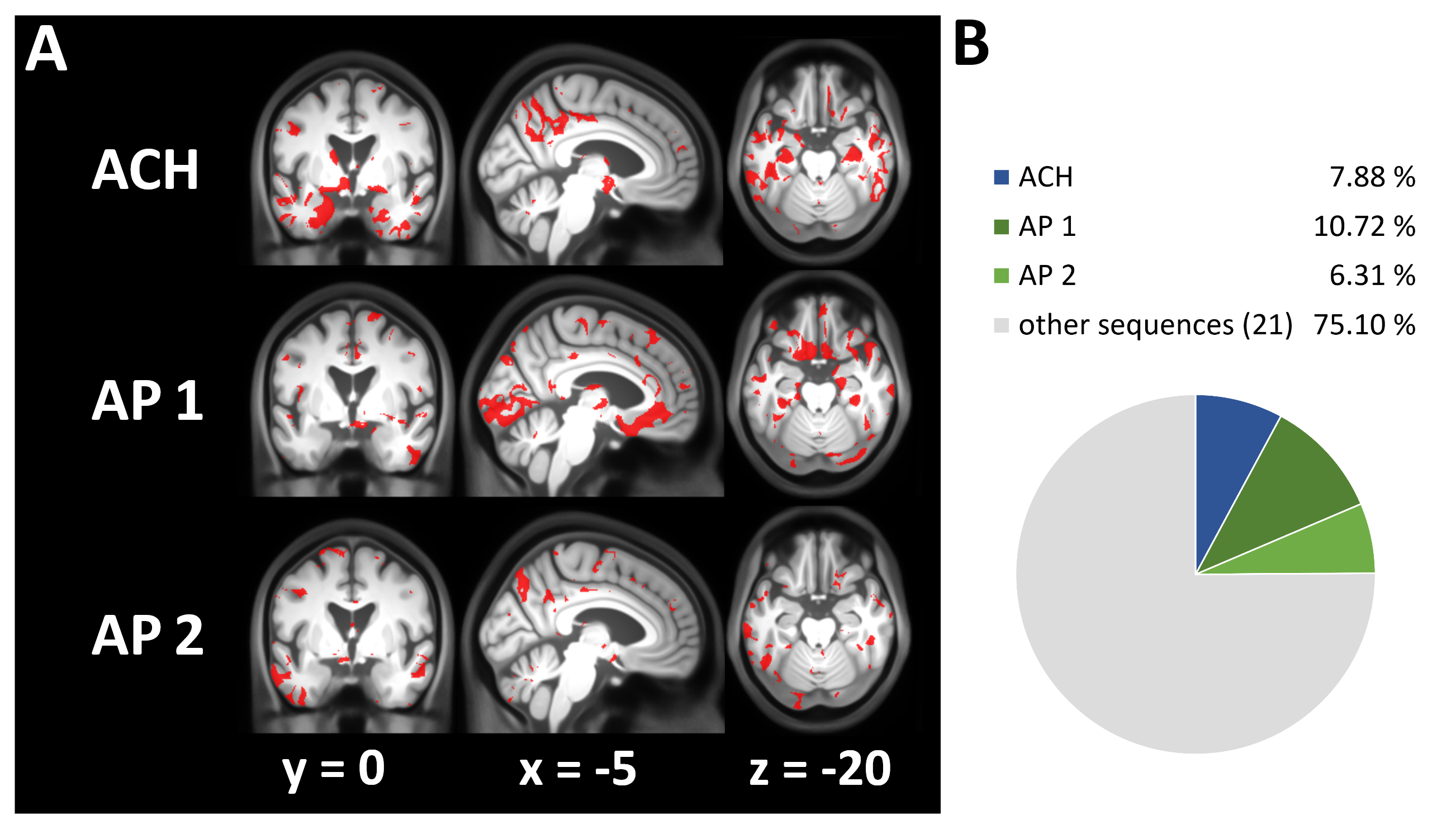

Supplement: Supplementary file 5 — Additional file 5. Face validity of ACH using VBM and CSF-total-Tau. Voxel-based evidence for monotonic volume decline over 24 sequences gained by permutation of the ACH sequence (ACH, A-T-N-➔A+T-N-➔A+T+N-➔A+T+N+); AP 1: A+T-N-➔A+T+N-➔A-T-N-➔A+T+N+; AP 2: A+T-N-➔A-T-N-➔A+T+N-➔A+T+N+; A: voxels where sequence shows highest evidence; B: percentage of gray matter voxels where sequence has highest evidence. Neurodegeneration (N) by CSF Total Tau. [file 13195_2023_1185_MOESM5_ESM.png]

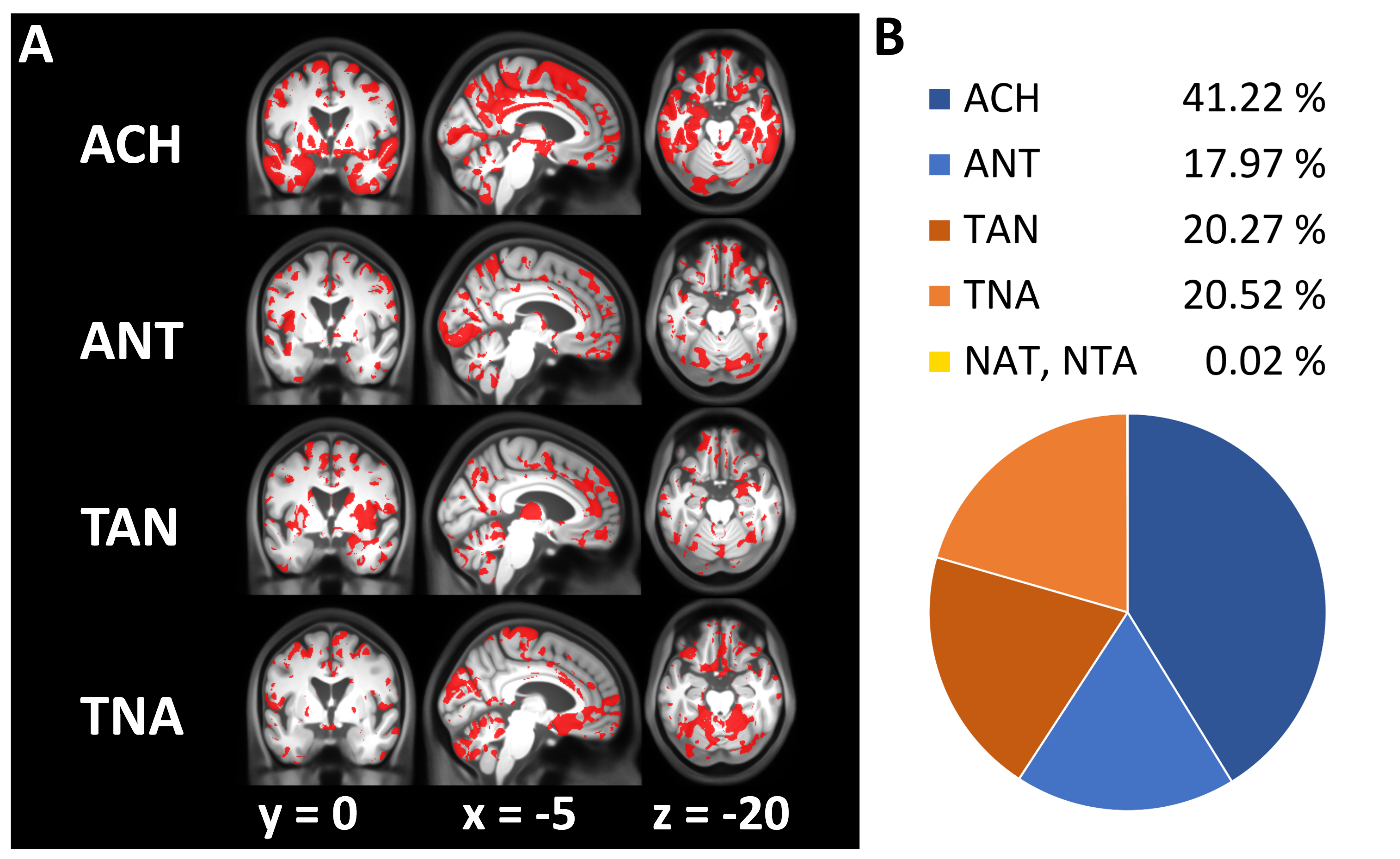

Supplement: Supplementary file 6 — Additional file 6. Comparing progression sequences towards AD pathology using VBM and CSF-total-Tau. Voxel-based evidence for monotonic volume decline over 6 possible sequences from A-T-N- towards A+T+N+ (ACH, ANT, TAN, TNA, NAT, NTA). Sequences are denoted in the order of biomarker positivity along the pathway (e.g. ANT = Amyloid-positivity first, Neurodegeneration second, Tau last). A: voxels where sequence shows highest evidence; B: percentage of gray matter voxels where sequence has highest evidence. N-first sequences (NAT, NTA) are not shown as only few voxels are supported. Neurodegeneration (N) by CSF Total Tau. [file 13195_2023_1185_MOESM6_ESM.png]
