# Supplementary material for: Exploring the ATN classification system using brain morphology
Source: Alzheimers Res Ther. 2023 Mar 13;15:50. doi: 10.1186/s13195-023-01185-x (PMC10009950; doi:10.1186/s13195-023-01185-x)
Supplement: Supplementary file 3 — Additional file 3. Distribution of ATN status and clinical diagnosis using CSF-total-Tau. Left: percentual distribution of selected ATN groups per clinical diagnosis; right: percentual distribution of clinical diagnosis per ATN groups. Neurodegeneration (N) by CSF Total Tau. [file 13195_2023_1185_MOESM3_ESM.pptx]

## Slide 1
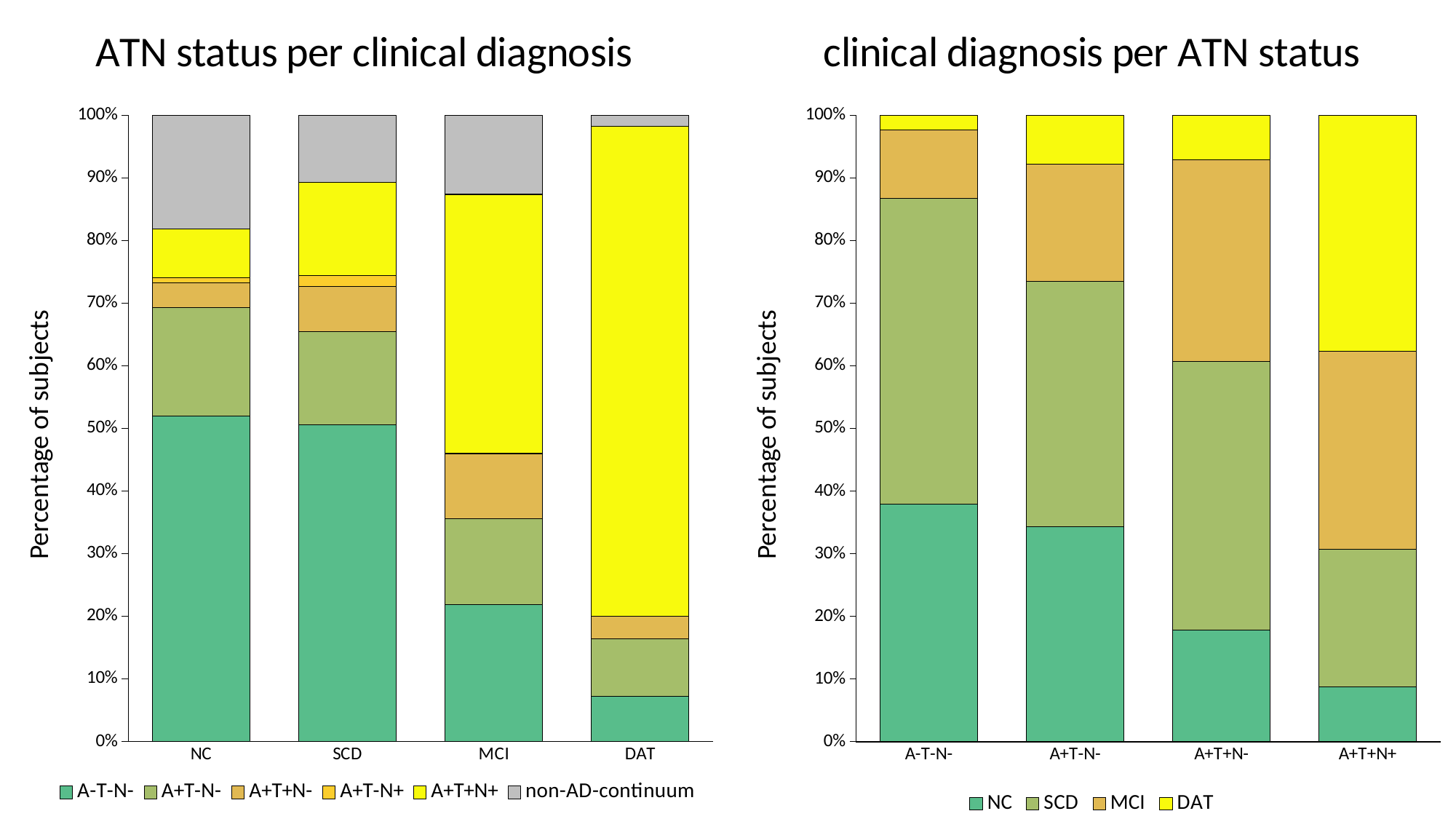

### Chart: ATN status per clinical diagnosis
| Category | A-T-N- | A+T-N- | A+T+N- | A+T-N+ | A+T+N+ | non-AD-continuum |
|---|---|---|---|---|---|---|
| NC | 66.0 | 22.0 | 5.0 | 1.0 | 10.0 | 23.0 |
| SCD | 85.0 | 25.0 | 12.0 | 3.0 | 25.0 | 18.0 |
| MCI | 19.0 | 12.0 | 9.0 | 0.0 | 36.0 | 11.0 |
| DAT | 4.0 | 5.0 | 2.0 | 0.0 | 43.0 | 1.0 |
### Chart: clinical diagnosis per ATN status
| Category | NC | SCD | MCI | DAT |
|---|---|---|---|---|
| A-T-N- | 66.0 | 85.0 | 19.0 | 4.0 |
| A+T-N- | 22.0 | 25.0 | 12.0 | 5.0 |
| A+T+N- | 5.0 | 12.0 | 9.0 | 2.0 |
| A+T+N+ | 10.0 | 25.0 | 36.0 | 43.0 |
